# Opposing roles of hematopoietic-specific small GTPase Rac2 and the guanine nucleotide exchange factor Vav1 in osteoclast differentiation

**DOI:** 10.1038/s41598-020-63673-6

**Published:** 2020-04-27

**Authors:** In Soon Kang, Jin Sun Jang, Chaekyun Kim

**Affiliations:** Laboratory of Leukocyte Signaling Research, Department of Pharmacology, Inha University School of Medicine, Incheon, 22212 Korea

**Keywords:** Cell biology, Immunology

## Abstract

Vav1 regulates Rac activation as a hematopoietic-specific Rho/Rac-family guanine nucleotide exchange factor. Rac is a subfamily of Rho GTPases that regulates the bone-resorbing capacity of osteoclasts (OCs). In this study, we show that hematopoietic-specific Rac2 and Vav1 play opposing roles by enhancing or attenuating OC differentiation, respectively. This was demonstrated by higher and lower bone density in the femurs from Rac2-deficient (Rac2^−/−^) and Vav1-deficient (Vav1^−/−^) mice, respectively, compared to the wild-type (WT) mice. Accordingly, Rac2^−/−^ cells displayed low numbers of tartrate-resistant acid phosphatase (TRAP)-positive multinucleated cells (41%) compared to WT cells, whereas, Vav1^−/−^ cells showed high TRAP-positive cell numbers (150%), and the double-knockout Rac2^−/−^Vav1^−/−^ mice nullified the effects on OC numbers achieved by the individual knockouts. These reciprocal roles of Rac2 and Vav1 in OC differentiation were confirmed by reduced and increased levels of OC-specific markers, such as TRAP, calcitonin receptor, cathepsin K, and DC-STAMP in the Rac2^−/−^ and Vav1^−/−^ OCs, respectively. Our findings of decrease and increase in actin ring formation and α_v_β_3_ integrin-mediated adhesion in Rac2^−/−^ and Vav1^−/−^ mice, respectively, suggest that Vav1 and its downstream GTPase, Rac2, may counteract to fine-tune OC differentiation and bone resorption.

## Introduction

Bone homeostasis depends on the balance between bone formation and resorption, wherein bone-forming osteoblasts and bone-resorbing osteoclasts (OCs) play central roles^1^. Osteoblasts/stromal cells play important roles during OC differentiation and activation by producing soluble factors, such as macrophage colony stimulating factor (M-CSF) and receptor activator of NF-κB ligand (RANKL)^2^. Although the initial steps in OC differentiation from OC precursors (pre-OCs) have not been defined completely, OC-like cells can be generated *in vitro* by treating bone marrow cells (BMCs) with M-CSF and RANKL whose receptors are c-Fms and RANK, respectively^3,4^. M-CSF and RANKL play distinct roles in multiple steps leading to OC maturation. M-CSF is involved mainly in the differentiation of hematopoietic stem cells into pre-OCs. M-CSF/c-Fms binding is responsible for the expression of RANK at the cell surface. RANKL/RANK signaling stimulates relatively downstream steps for inducing the formation of multinucleated mature OCs. RANKL is responsible for inducing OC-specific target genes including NFATc1, the master transcriptional regulator of OC differentiation^5^. Genetic deletion and/or inactivation of either M-CSF or RANKL result in complete absence of mature OCs accompanied by abnormally dense bone^6,7^. Mature OCs are multinucleated cells formed by the cell fusion of pre-OCs originated from hematopoietic progenitors of the monocyte/macrophage lineage. The cell fusion involves multiple steps such as migration/chemotaxis of pre-OCs into close proximity which is associated with dynamic actin rearrangement for cellular movement. Such actin rearrangement is dependent on attachment to the matrix via α_v_β_3_ integrin, the major integrin in OCs which recognizes ligands such as vitronectin^8^. The mature multinucleated OCs resulted from cell fusion resorb mineralized bone tissues^4^.

Rac is a subfamily of Rho family GTPases with three isoforms, Rac1, Rac2, and Rac3, which share high sequence homology. Rac1 and Rac3 are expressed in a wide variety of tissues, whereas Rac2 is expressed in hematopoietic cells, including OCs. Rac isoforms play distinct cell type-specific functions. Rac GTPases are crucial not only in OC differentiation and function but also in OC survival intimately involving in actin rearrangement for cellular migration/chemotaxis^9–12^. For instance, Rac1 plays a significant role in regulating the OC apoptosis and motility^11^. Rac2 is also known to involve in OC differentiation by the evidence that pre-OCs lacking Rac2 are defective for chemotaxis and resorptive activity^12^. Furthermore, it was reported that both Rac1 and Rac2 are required for optimal OC with non-overlapping roles during osteoclastogenesis^13^. This was supported by others^14^ who demonstrated that Rac1-deficient mice produced severely reduced numbers of OCs while Rac2-deficient mice generated normal numbers of OCs but impaired for full differentiation. These data implicate that Rac1 and Rac2 are involved in OC differentiation with different roles. More recent study indicated that Rac1 or Rac2 alone had no effect on osteoclastogenesis but Rac1 and Rac2 were mutually compensatory for cytoskeleton organization and generation of the key resorptive organelles in OC^15^. In addition to Rac GTPases, Cdc42, a sister GTPase, also mediates bone resorption by stimulating osteoclastogenesis^16^. Although numerous studies demonstrated that Rac GTPases are important for regulating OC differentiation, there have been many conflicting reports on the roles of Rac GTPase subsets in OC differentiation and therefore relative functional roles of Rac GTPases remain to be determined.

ROS are an important component associated with generation of OCs and bone resorption^17^. The main source of ROS in OCs is NADPH oxidase (NOX), which consists of two integral membrane proteins (gp91^phox^ and p22^phox^) and four cytosolic subunits (p67^phox^, p47^phox^, p40^phox^ and Rac)^18^. The common roles of Rac1 and Rac2 is to regulate NOX responsible for generating ROS. Previously we have demonstrated that Rac2 is required for NOX2 assembly which in turn plays a role in chemotactic movement of the neutrophil^19^. Since Rac2 is expressed predominantly in the monocyte/macrophage lineage including neutrophil, and neutrophils are derived from the myeloid granulocyte progenitors, the same lineages which OCs are derived from, we investigated whether any downstream signaling pathways of Rac2 are associated with OC differentiation. During these studies, we found that the downstream signaling product of Rac2, gp91^phox^/NOX2-derived ROS was important for promoting efficient OC differentiation by inducing NFATc1 as a downstream signaling mediator of RANK^20^.

One of the upstream activation signaling pathway of Rac2 is the guanine nucleotide exchange factor (GEF), Vav1. In contrast to Vav2 and Vav3 found broadly in all tissues, Vav1 is expressed predominantly in hematopoietic cells. Recently, we have found that Vav1 inhibits OC differentiation and protects against bone resorption^21^. Because Rac2 is a direct downstream of Vav1, we extended our study to see whether Rac2 would act to or inhibit OC differentiation similarly to Vav1 or promote OC differentiation as reported by others^13–15^. We also wanted to see whether the effect of Rac2 on OC differentiation would be compromised by the absence of Vav1. To this end, we employed a genetic approach using Vav1- and Rac2-deficient mice (Vav1^−/−^ and Rac2^−/−^, respectively) to analyze the relationship between Vav1 and Rac2 roles in OC differentiation. We found that while Vav1 deficiency enhanced OC differentiation, Rac2 deficiency inhibited OC differentiation indicating the active role of Rac2 in OC differentiation. Moreover, Vav1 deficiency could fully restore the impaired OC differentiation resulted from Rac2 deficiency, which suggests that unknown stimulatory pathways other than Rac2 is activated by Vav1 deficiency. Interestingly the Vav1^−/−^ mice showed significantly elevated Vav3 expression suggesting that Vav1 deficiency may induce Vav3 as a compensatory response which promotes OC differentiation in a Rac2-independent manner.

## Results

### Vav1^−/−^ and Rac2^−/−^ BMCs show enhanced and attenuated OC differentiation capacity, respectively, during *in vitro* differentiation

Rac GTPases control many aspects of cell behavior through the regulation of multiple signal transduction pathways. Rac proteins act as binary switches by cycling between an inactive (GDP-bound) and active (GTP-bound) conformational state. Vav is a GEF responsible for activating Rac GTPases through exchanging GDP to GTP. We have previously demonstrated that Vav1^−/−^ mice show excessive bone resorption, resulting in low bone density in the femurs and suggesting Vav1 as a suppressive factor for OC differentiation^21^. In this study, we extended our research to examine whether Rac2 also suppresses OC differentiation as a hematopoietic-specific downstream effector of Vav1 using Vav1^−/−^ and Rac2^−/−^ mice.

**Figure 1:**
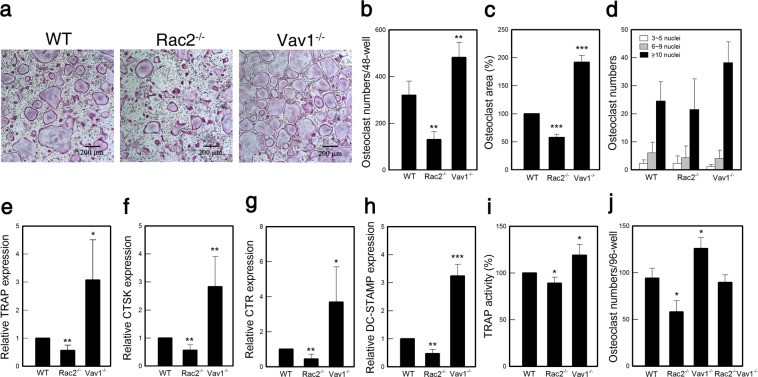
Rac2 enhanced but Vav1 suppressed OC differentiation. (**a,b**) BMCs were differentiated into OCs with 30 ng/mL M-CSF and 50 ng/mL RANKL. Cells were fixed with 10% formalin, permeabilized with 0.1% Triton X-100, and stained with TRAP solution. TRAP-positive multinucleated cells (≥3 nuclei) were counted as OCs (n = 10 independent experiments, each performed in triplicate). (**c**) OC areas relative to WT were determined using ImageJ software (n = 4), and (**d**) the number of OCs with respect to nuclear numbers per cell were counted in selected fields (n = 4). (**e–h**) The mRNA expressions of OC marker genes, such as TRAP, CTSK, CTR, and DC-STAMP were determined by using qRT-PCR. GAPDH mRNA expression level was used to ensure equal loading (n = 6). (**i**) TRAP enzyme activity was determined in OCs (n = 4). (**j**) OC numbers from Rac2^−/−^Vav1^−/−^ double-KO mice were determined (n = 3 independent experiments, each performed in triplicate). *p < 0.05, **p < 0.01, and ***p < 0.001 compared to WT.

To study whether Rac2 inhibits OC differentiation similarly to Vav1 or promotes OC differentiation, we determined OC differentiation. Using the BMCs from Vav1^−/−^ and Rac2^−/−^ mice cultured under differentiating conditions with M-CSF and RANKL, we probed tartrate-resistant acid phosphatase (TRAP) expression, a canonical marker for mature OC, through histochemical analysis. The numbers of multinuclear TRAP-positive cell populations in the differentiating OCs were measured. Rac2^−/−^ cells displayed low numbers of TRAP-positive multinucleated cells (41%) compared to wild-type (WT) cells, whereas, Vav1^−/−^ cells showed high TRAP-positive cell numbers (150%) (Fig. 1a,b). OC size and nucleus number per cell were consistent with OC numbers (Fig. 1c,d). We then compared the enzymatic activities of TRAP in differentiating cells from Vav1^−/−^ and Rac2^−/−^ mice *in vitro*. Consistent with OC numbers, Rac2^−/−^ and Vav1^−/−^ cells exhibited relatively lower and higher TRAP activities, respectively, than WT cells (Fig. 1i).

Because our results show the opposing roles of Vav1 and Rac2 in *in vitro* OC differentiation by M-CSF and RANKL, we aimed to determine how BMCs from Rac2^−/−^Vav1^−/−^ double-knockout (KO) mice would behave in OC differentiation *in vitro*, especially in comparison to the BMCs from Rac2^−/−^ and Vav1^−/−^ single-KO mice. Results indicated that Rac2^−/−^Vav1^−/−^ double-KO cells contained TRAP-positive cell populations at intermediate levels between those in Rac2^−/−^ and Vav1^−/−^ single-KO cells (Fig. 1j and Supplementary Fig. S1). These data suggest that Rac2 and Vav1 may play the opposing rather than equivalent roles in OC differentiation.

### Vav1^−/−^ and Rac2^−/−^ OCs express OC-specific genes at higher and lower levels, respectively, during *in vitro* differentiation

To confirm our finding of the contrasting roles of Rac2 and Vav1 in OC differentiation, we used quantitative RT-PCR (qRT-PCR) to compare the mRNA expression levels of TRAP and other OC markers, such as cathepsin K (CTSK), calcitonin receptor (CTR), and dendritic cell specific transmembrane protein (DC-STAMP), in Rac2^−/−^ and Vav1^−/−^ cells after differentiation with M-CSF and RANKL. All OC markers showed relatively low and high expression levels in Rac2^−/−^ and Vav1^−/−^ cells, respectively, compared to WT cells (Fig. 1e–h). These results supported our hypothesis that Rac2 and Vav1 play positive and negative roles in OC differentiation, respectively.

### Vav1^−/−^ and Rac2^−/−^ pre-OCs show higher and lower adhesion activity to vitronectin and actin ring formation, respectively, during *in vitro* differentiation

**Figure 2:**
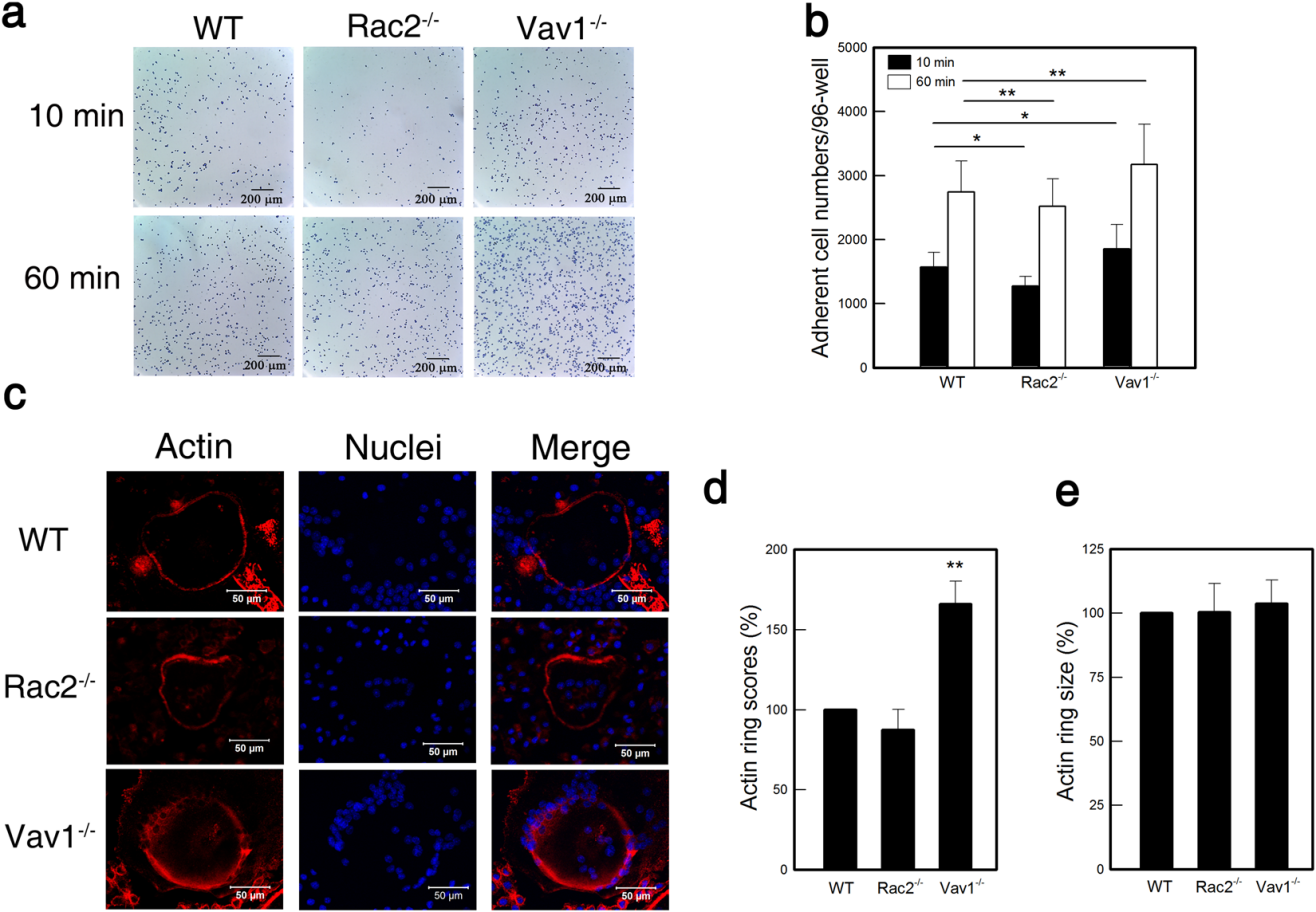
Rac2 increased but Vav1 inhibited adhesion to vitronectin and actin ring formation of OCs. (**a**) OC precursor cells from Rac2^−/−^, Vav1^−/−^, and WT mice were placed on vitronectin-coated plates for 10 and 60 min at 37 °C, and the ability of pre-OCs to adhere to vitronectin was determined. (**b**) Bar graphs showed numbers of adhered cells at 10 and 60 min (n = 4) (**c**) Actin ring formation of OCs plated on glass was determined under fluorescence microscopy. F-actin and nuclei were stained by Alexa-phalloidin (red) and DAPI (blue), respectively (n = 3). (**d**) The fluorescence intensity of actin ring, and (**e**) actin ring size of 12 cells per experiment were analyzed by using ImageJ software. *p < 0.05 and **p < 0.01 compared to WT.

Integrins are heterodimeric adhesion receptors that mediate bone resorption processes, including various cell-cell and cell-matrix interactions. OCs express the α_v_β_3_ integrin, which binds to a variety of extracellular matrix proteins, including vitronectin, during bone resorption^22^. Rac and Vav are known to control integrin-mediated cell adhesion. Thus, we supposed that Rac2 and Vav1 deficiencies would affect the α_v_β_3_-mediated adhesion to vitronectin. To determine the effect of Rac2 and Vav1 on α_v_β_3_-mediated adhesion, Pre-OCs from Rac2^−/−^, Vav1^−/−^, and WT mice were placed on vitronectin-coated plates for 10 and 60 min at 37 °C, and the adhered cells were washed and counted (Fig. 2a,b). Rac2^−/−^ cells showed significantly defective adhesive activity to vitronectin (p = 0.011 at 10 min and p = 0.046 at 60 min) (Fig. 2b). In contrast, Vav1^−/−^ cells possessed stronger adhesive activity than WT cells. Thus, Rac2 and Vav1 were shown to play opposing roles through their respective positive and negative effects on α_v_β_3_-mediated adhesion to vitronectin. These results were consistent with our previous findings regarding the effects of Rac2 and Vav1 on OC-specific marker expression in the BMCs following differentiation into OCs.

OC adhesion to the bone during bone resorption leads to actin ring formation, a prerequisite for OC bone resorption. Rac and Rho GTPases are known to regulate actin ring formation and lacunae resorption in OCs^23^. Thus, we explored the possibility that Rac2 and Vav1 might affect actin ring formation in the BMCs following differentiation into OCs through staining F-actin with Alexa-phalloidin. Consistent with our earlier results, Rac2^−/−^ OCs showed slightly reduced (p = 0.521) and Vav1^−/−^ OCs showed significantly increased (p = 0.002) actin ring formation compared to WT cells (Fig. 2c,d). However, little difference was detected in actin ring sizes (Fig. 2e).

### Femurs from Vav1^−/−^ and Rac2^−/−^ mice contain higher and lower numbers of TRAP-positive cells, respectively

**Figure 3:**
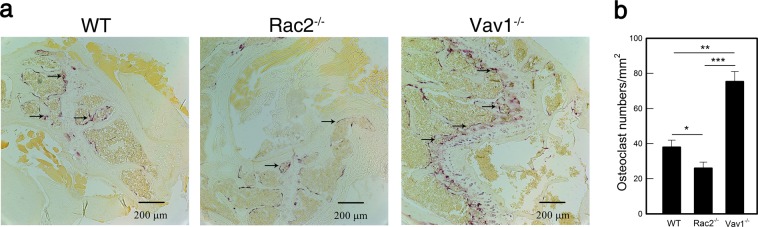
TRAP-positive multinucleated cell population was decreased Rac2^−/−^ femurs but increased in Vav1^−/−^ femurs. (**a**) Histological sections of femurs from 6-wk-old male mice were stained for TRAP activity and counterstained with hematoxylin (n = 3). (**b**) OC numbers from femurs per selected field were counted. *p < 0.05, **p < 0.01, and ***p < 0.001 compared to WT.

So far, all experiments were conducted using BMCs differentiating into OCs in the presence of M-CSF and RANKL. To determine whether the *in vitro* OC defects were reflective of an *in vivo* defect, we examined the femurs from Rac2^−/−^ and Vav1^−/−^ mice *in situ* to investigate the effects of Rac2 and Vav1 on OC differentiation. To this end, the femurs from Rac2^−/−^ and Vav1^−/−^ mice were sliced and stained for TRAP, followed by histological counterstaining with hematoxylin. Compared to WT mice, femoral sections from Rac2^−/−^ mice displayed 68% TRAP-positive cells, whereas Vav1^−/−^ mice showed twice as many (197%) (Fig. 3). In agreement with our earlier results obtained from *in vitro* experiments, the data obtained from the femurs clearly indicated that Rac2 and Vav1 were involved in OC differentiation in opposing manners through enhancing and suppressing OC differentiation, respectively.

### Rac2 and Vav1 may play opposing roles in deteriorating and protecting bone structure, respectively

**Figure 4:**
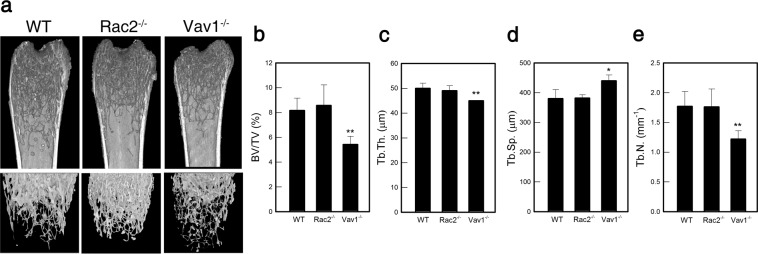
Rac2 deteriorated but Vav1 protected bone structure of the femurs. (**a**) Microcomputed tomography of the femurs of WT, Rac2^−/−^ and Vav1^−/−^ mice were performed. (**b–e**) Bone structural parameters, such as BV/TV, trabecular thickness (Tb.Th.), and trabecular separation (Tb.Sp.) were increased in Rac2^−/−^ mice whereas those were decreased in Vav1^−/−^ mice (n = 4). *p < 0.05 and **p < 0.01 compared to WT.

Finally, we determined the bone densities from Rac2^−/−^ and Vav1^−/−^ mice *in situ* using μCT. The images clearly showed dense femurs in Rac2^−/−^ mice compared to WT mice, whereas, those from Vav1^−/−^ mice showed severe bone resorption (Fig. 4a)^21^. These data were consistent with our earlier results that Rac2 and Vav1 may have opposing roles by promoting and inhibiting OC differentiation, respectively. To verify these results, we further analyzed several bone structural parameters used to represent trabecular bone architecture^24^, namely, the bone volume fraction (BV/TV), trabecular thickness (Tb.Th.), trabecular separation (Tb.Sp.), and trabecular number (Tb.N.). Compared to WT mice, the parameters indicative of healthy bone structure were not changed in the femurs from Rac2^−/−^ mice, whereas those in Vav1^−/−^ femurs were significantly low (p = 0.006 in BV/TV; p = 0.003 in Tb.Th.; p = 0.006 in Tb.N.) (Fig. 4b,c,e) and trabecular separation was significantly high (p = 0.014 in Tb.Sp.) (Fig. 4d). Taken together, these data lead us to conclude that Rac2 may act to deteriorate the bone architecture by promoting OC differentiation, whereas Vav1 may protect the bones from resorption by suppressing OC differentiation.

### Expression of Vav3, which known to promote OC differentiation, is elevated by Vav1 deletion

**Figure 5:**
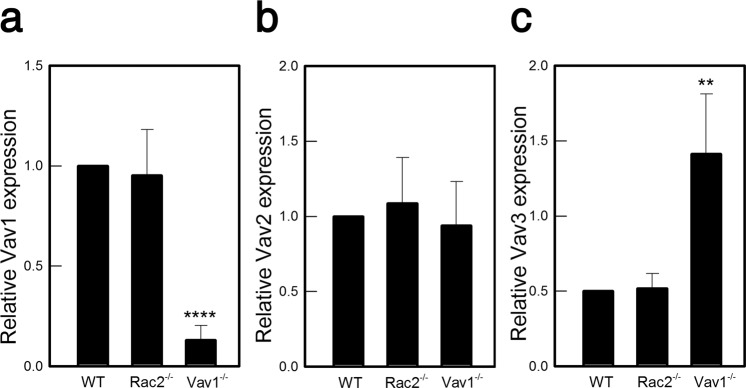
Expression of Vav3 was increased in Vav1^−/−^ OCs. (**a–c**) The mRNA expression of Vav isoforms was determined by qRT-PCR. The Vav1 mRNA expression (**a**) was negligible in Vav1^−/−^ OCs (p < 0.0001) and Vav3 mRNA expression (**c**) was significantly increased (p = 0.007) (n = 5). GAPDH mRNA expression level was used to ensure equal loading.

Unlike Vav1, Vav3 has been reported to play an enhancing role in OC differentiation. This was demonstrated previously, as Vav3-deficient mice had increased bone mass and were protected from bone loss induced by systemic bone resorption stimuli, such as parathyroid hormone or RANKL^25^. Thus, we examined the possibility that the expression of other Vav isoforms, such as Vav3, might be increased in the Vav1^−/−^ mice. We found that Vav3 mRNA levels were increased by approximately 2.5-folds in Vav1^−/−^ OCs compared to those in the WT mice (Fig. 5). Thus, it is conceivable that the poor bone density detected in the femurs of Vav1^−/−^ mice might be due to compensatory induction of Vav3, which, unlike Vav1, acts to enhance OC differentiation.

## Discussion

Rac GTPases are known to influence OC differentiation, but last several decades, many conflicting results have been reported about the specific roles of Rac in association with Vav subsets. This is mainly due to poorly identified upstream and downstream effectors of Rac GTPases involved in OC differentiation. In this study, we have demonstrated that Rac2^−/−^ BMCs were defective for M-CSF/RANKL-mediated OC differentiation *in vitro* and Rac2^−/−^ mice contained femurs with abnormally dense bones indicating the essential role of Rac2 in OC differentiation. In contrast, BMCs deficient of Vav1, a GEF responsible for activating Rho GTPases, differentiated into OC more effectively compared to WT cells. This is consistent with our previous finding that Vav1 acted to inhibit M-CSF/RANKL-mediated OC differentiation and protected bone from resorption^21^. Such opposing roles of Vav1 and Rac2 in OC differentiation seem incompatible considering that Vav1 resides on the same line of signal transduction pathways presumably as an upstream activator of Rac2. So we investigated the issue to clarify whether Rac2 plays a positive role for OC differentiation both *in vitro* and *in vivo* experimental settings, and whether Vav1 the presumptive upstream activator of Rac2, affects the Rac2 effects on OC differentiation. In this study, we did not deal with Rac1 because Rac1 is ubiquitously expressed and is likely involve in NOX activation in non-hematopoietic cells.

Rac2 is known to be expressed preferentially in hematopoietic cells. We have previously described that Rac2 was required for activating NOX2 activation along with cell migration/chemotaxis and ROS production in neutrophils^19^. As a follow up study, we demonstrated that OC differentiation required NOX2-derived ROS via induced NFATc1 as a downstream signaling mediator of RANK^20,26^. The highlight findings of this study are as follow: (1) Rac2 plays a positive role for OC differentiation as evidenced by observations that Rac2^−/−^ BMCs were impaired for OC differentiation *in vitro* experimental settings. (2) The results from *in vitro* setting were confirmed by the *in vivo* mouse model where Rac2^−/−^ mice resulted in the slightly denser bones in the femurs. (3) The impaired OC differentiation resulted from Rac2 deficiency was restored to the normal levels by the depletion of Vav1. We hypothesized that Vav1 may play an inhibitory role in OC differentiation by suppressing certain channels required for OC differentiation and Vav1 deficiency may relieve the inhibitory blockade inducing other stimulatory Vav subsets which can promote OC differentiation in a Rac2-independent manner. We tested this possibility by comparing the expression levels of Vav1, Vav2, and Vav3 in the OCs derived from WT, Rac2^−/−^ and Vav1^−/−^ mice. Our results indicated that expression level of Vav3 was greatly increased by the depletion of Vav1, whereas there was no such increase in the Vav2 expression. Vav3 has been reported to play a stimulatory role in OC differentiation as evidenced by that Vav3-deficient mice showed increased bone mass and were protected from bone loss^25^. Our results suggest that Vav1 may control the Vav3 functions for OC differentiation by modulating the expression of Vav3. It is also possible that Vav3 may control the expression of Vav1 vice versa. We do not know whether Vav1 and Vav3 target identical Rac subsets to determine differential effects on OC differentiation or share the common downstream targets competing for the active sites. In any event, there may be functional regulatory communications among the Vav to affect the Rho GTPase-mediated OC differentiation. Our findings provide important clues for better understanding the complexity of the coordinated expression of Vav family members to modulate OC differentiation.

A feature of OC formation involves multiple fusions of pre-OCs derived from the monocyte-macrophage lineage cells. The processes require dynamic actin cytoskeleton to modulate the adhesion of OCs to bone and its subsequent resorption. Most information about the roles of Rho GTPases in OC differentiation has been acquired from mouse experimental models. The role of Rac2 in OC differentiation was supported by the studies with human subjects. Shwachman-Diamond syndrome (SDS) is resulted from mutations in the *SBDS* gene, characterized by neutrophil dysfunction and defective RANKL-mediated upregulation of Rac2^27^. Interestingly, a hallmark feature of SDS is impaired OC formation. The studies have shown that reduced Rac2 was causative for the clinical symptoms. They concluded that Filamin A and SBDS were required for Rac2-mediated OC differentiation by regulating monocyte migration. At this time, we do not know whether these human data are relevant with the phenomena seen in Rac2^−/−^ mice that we have observed.

In summary, we have demonstrate that Rac2 is required for the organization of the actin cytoskeleton and resorptive activity as evidenced by that actin ring formation and cell adhesion to integrin were severely impaired in the Rac2^−/−^ OCs. These results was confirmed by the *in vivo* observations that Rac2^−/−^ mice displayed the denser bones in the femurs compared to WT mice. The impaired OC differentiation resulted from Rac2 deficiency was fully restored by depletion of Vav1. The mice deficient of Vav1 increased expression of Vav3 implicating a suppressive role of Vav1 in Vav3 expression as well as functions. We tentatively conclude that the induced Vav3 resulted from Vav1 deficiency may activate Rho GTPases other than Rac2 promoting OC differentiation. These data suggest the complexity of the cross-talk between regulatory Vav GEF families for the Rac2-mediated OC differentiation modulating bone homeostasis or pathologic bone remodeling. The nature of these signaling links between Vav1 and Vav3 remains to be determined. Identification of these stimulatory and inhibitory Vav GEFs in Rac2-mediated OC differentiation will improve our understanding of how bone modeling is regulated and this information may help to treat osteolytic pathologies.

## Methods

### Reagents and antibodies

M-CSF and RANKL were purchased from PeproTech (London, UK). Cell culture reagents were purchased from Hyclone. Oligonucleotide primers for PCR were bought from Bioneer (Daejeon, Korea). SYBR Green was purchased from KAPA Biosystems. Antibody against Rac2 was purchased from Upstate, and Rac1 antibody and horseradish peroxidase-conjugated mouse- and rabbit-IgG antibodies were obtained from BD Pharmingen. Other chemicals were purchased from Sigma unless indicated otherwise.

### Mice

Vav1^−/−^ mice and Rac2^−/−^ mice were generated previously^28,29^, Rac2^−/−^Vav1^−/−^ double-KO mice were generated by crossing Vav1^−/−^ and Rac2^−/−^ mice, and C57BL/6J mice purchased from Jackson Laboratory were used for WT controls. Mice were housed under pathogen-free conditions at the animal facility of Inha University. All procedures were conducted in accordance with the institutional guidelines approved by the Animal Care and Use Committee of Inha University.

### OC differentiation from BMCs

Murine OCs were differentiated from BMCs as described previously^20^. BMCs were prepared from 6~10-wk-old male mice and cultured overnight in α-MEM supplemented with 10% FBS and 10 ng/mL M-CSF. Non-adherent BMCs were collected the next day, and cultured continuously with 30 ng/mL M-CSF for more 3 days to induce differentiation into OC precursor cells. These cells were cultured for an additional 4 days in the presence of 30 ng/mL M-CSF and 50 ng/mL RANKL. Functional OC formation was assessed by counting the number of TRAP-positive cells containing more than three nuclei. OC size was determined using ImageJ software (NIH) and number of nuclei per OC was counted.

### TRAP staining

TRAP-positive cells were stained by using a leukocyte acid phosphatase assay kit (Sigma)^20^. Briefly, cells were washed with PBS and fixed using a fixative solution containing citrate solution, acetone, and 37% formaldehyde. Fixed cells were treated with TRAP staining solution for 60 min at 37 °C and washed twice with water. After counterstaining with hematoxylin, the number of TRAP-positive cells containing three or more nuclei was counted.

### TRAP activity

TRAP activity was determined as described by Kang and Kim^20^. Briefly, cells were incubated with 10 mM sodium tartrate and 5 mM p-nitrophenyl phosphate in 50 mM citrate buffer, pH 4.5 for 30 min at 37 °C. The enzyme reaction was then terminated with 0.1 N NaOH. Absorbance was measured at 405 nm using a VersaMax microplate reader (Molecular Devices) equipped with SoftMax software.

### qRT-PCR

Total RNA was extracted from the OCs using TRI reagent, and then reverse transcribed according to the protocol provided by Takara Bio (Tokyo, Japan). Then, qRT-PCR was carried out on an Applied Biosystems StepOne unit using SYBR Green PCR Master Mix (Toyobo, Osaka, Japan) and the following primers (forward and reverse, respectively): Vav1, 5′-CTA CGG GAT CTG CTG ATG GT-3′ and 5′-CTG CCG TAG GGT TTC ATT GT-3′; Vav2, 5′-CAG ACG GAC AAT CTG CTC AA-3′ and 5′-CCA ACT GCT TGA AGC TTT CC-3′; Vav3, 5′-ACC TTC ACT CGG GTG ACA TC-3′ and 5′-TCC ACC TGT CTG GAA GCT CT-3′; TRAP, 5′-ACG GCT ACT TGC GGT TTC A-3′ and 5′-TCC TTG GGA GGC TGG TCT T-3′; CTSK, 5′-GAA GAA GAC TCA CCA GAA GCA G-3′ and 5′-TCC AGG TTA TGG GCA GAG ATT-3′; CTR, 5′-TGC TGG CTG AGT GCA GAA ACC-3′ and 5′-GGC CTT CAC AGC CTT CAG GTA C-3′; DC-STAMP, 5′-TCC TCC ATG AAC AAA CAG TTC CAA-3′ and 5′-AGA CGT GGT TTA GGA ATG CAG CTC-3′; GAPDH, 5′-CCT TCC GTC CTA CCC C-3′ and 5′-CCC AAG ATG CCC TTC ATG-3′.

### Adhesion assay

OC precursor cells (5 × 10^4^) were seeded in 48-well plates coated with 10 μg/mL vitronectin (R&D Systems) for 10 and 60 min at 37 °C. Cells were rinsed with PBS to remove unattached cells, stained with Diff-Quick solutions (Thermo), and counted to determine the adhered cell numbers.

### Histology and microcomputed tomography (μCT) analysis

OCs on sterile cover glass were fixed and F-actin and nucleus were stained with 0.66 μM Alexa-phalloidin (Thermo) and 0.5 μg/ml DAPI, respectively, for 45 min in the dark at room temperature. Cells were washed with PBS twice and observed under the fluorescence microscopy (Zeiss, Germany)^21^. To quantify the extent of actin ring formation, phalloidin-stained OC images were uploaded into ImageJ software and total red channel fluorescent density was calculated by selecting the entirety of the cell.

The femurs of 6-wk-old male mice were fixed with 10% formalin, decalcified with 10% EDTA, dehydrated, embedded in paraffin, and sliced into 5 μm sections. The sections were stained either for TRAP or with hematoxylin and eosin. The trabecular microarchitecture of the distal femoral metaphysis was determined using SkyScan μCT (Brucker, Kontich, Belgium) at 80 kV, 80 μA, and a 6.5-μm voxel size. Cortical bone parameters including the ratio of trabecular bone volume to total bone volume (BV/TV, %), trabecular thickness (Tb.Th., μm), trabecular separation (Tb.Sp., μm), and the number of trabeculae (Tb.N., mm^−1^) were analyzed by μCT at 3000 ms per projection (500 projections in total)^20^.

### Statistical analyses

Two-tailed Student’s *t*-test (paired) was performed using Microsoft Excel. Data are expressed as the mean ± SD or mean ± SEM (Fig. 2d), and a p value of less than 0.05 was considered statistically significant.

## Supplementary Information

Supplementary Information.

## Data Availability

The datasets generated during the current study are available from the corresponding author on reasonable request.
