# Supplementary figures and images for: Opposing roles of hematopoietic-specific small GTPase Rac2 and the guanine nucleotide exchange factor Vav1 in osteoclast differentiation
Source: Sci Rep. 2020 Apr 27;10:7024. doi: 10.1038/s41598-020-63673-6 (PMC7184755; doi:10.1038/s41598-020-63673-6)

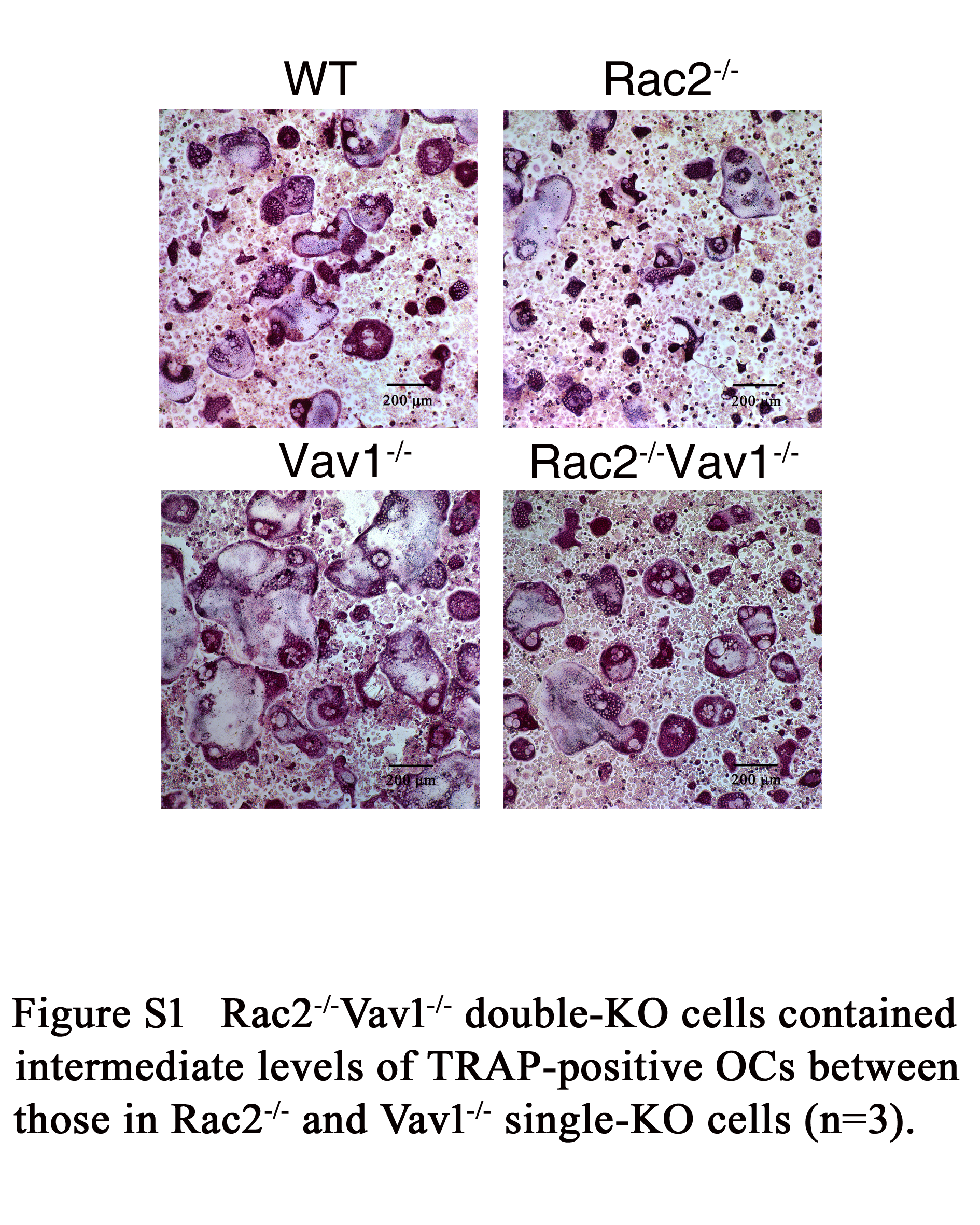

Supplement: Supplementary file 1 — Supplementary Information. [file 41598_2020_63673_MOESM1_ESM.tif]
